# Surgical Management in a Paediatric Case of Endobronchial Mucoepidermoid Carcinoma Involving the Carina

**DOI:** 10.7759/cureus.48680

**Published:** 2023-11-12

**Authors:** Mark A Boyle, Oloruntobi Rotimi, Abigail Palmares, Jose Alvarez Gallesio, Abdullah Alshammari, Thomas Semple, Silviu Buderi, Simon Jordan

**Affiliations:** 1 Department of Surgery and Cancer, Imperial College London, London, GBR; 2 Department of Thoracic Surgery, Royal Brompton Hospital, London, GBR; 3 Department of General Surgery, Medway NHS Foundation Trust, Gillingham, GBR; 4 Department of Radiology, Royal Brompton Hospital, London, GBR

**Keywords:** paediatric surgery, lobectomy, carinal resection, thoracotomy, endobronchial tumour, thoracic surgery, mucoepidermoid cancer

## Abstract

Although mucoepidermoid carcinoma (MEC) is the most diagnosed malignancy of the salivary gland, it rarely localises to the bronchus, accounting for only 0.1-0.2% of all primary lung malignancies. Of those pulmonary MECs, most are found in segmental or lobar bronchi, and they are rarely found in mainstem bronchi, highlighting the novelty of this presentation for thoracic specialists. We present a case report of a seven-year-old female who underwent a carinal resection and a right upper lobectomy for the management of an endobronchial MEC causing right middle lobe (RML) obstruction. Intraoperatively, an exophytic mass originating from the junction of the right main bronchus and bronchus intermedius was identified, causing a partial obstruction of the RML bronchus. Frozen sections demonstrated clear margins and follow-up bronchoscopies have been unremarkable. Given their rarity, endobronchial MECs can be diagnostically difficult and cause uncertainty with respect to their management. Low-grade tumours have a much more favourable prognosis than their high-grade counterparts, with surgical resection being the gold standard of care. Therefore, the index of suspicion, time to diagnosis, and definitive treatment are critical to the outcome.

## Introduction

Endobronchial mucoepidermoid carcinoma (MEC) is a rare malignant tumour occurring in the glandular tissue of the bronchial submucosa and accounts for 0.1-0.2% of all primary lung cancers [1,2]. It is characterised by a mixture of mucus-secreting, squamous epithelial and intermediate cells within the tumour [3,4]. Given their rarity, there have been differences of opinion regarding the aggressiveness and subsequent management of these tumours. It appears they display a continuum of virulency with low-grade lesions amenable to surgical cure but higher-grade lesions proving to be challenging [3].

In the paediatric population, endobronchial MEC is known to cause airway obstruction, resulting in symptoms such as cough, dyspnoea, recurrent respiratory infections, and wheezing. The nonspecificity of these symptoms frequently results in delays or misdiagnosis; however, time is critical to offer a potential surgical cure [5]. Herein, we present the case of a seven-year-old girl who underwent successful surgical management for endobronchial MEC in an effort to add to the growing body of literature on this potentially curable presentation.

## Case presentation

A seven-year-old Caucasian girl, with no previous medical history of note, presented to her local emergency department with fevers, cough, wheezing, and weight loss, having presented with the same symptoms five months prior. At the time of her initial presentation, a chest X-ray (CXR) demonstrated some diffused opacity in the right lower zone of her lung, and blood results revealed a raised C-reactive protein (CRP) of 82 mg/L. Although blood cultures later returned as negative, the patient was diagnosed with right lower lung pneumonia and was treated with a two-week course of oral antibiotics.

**Figure 1:**
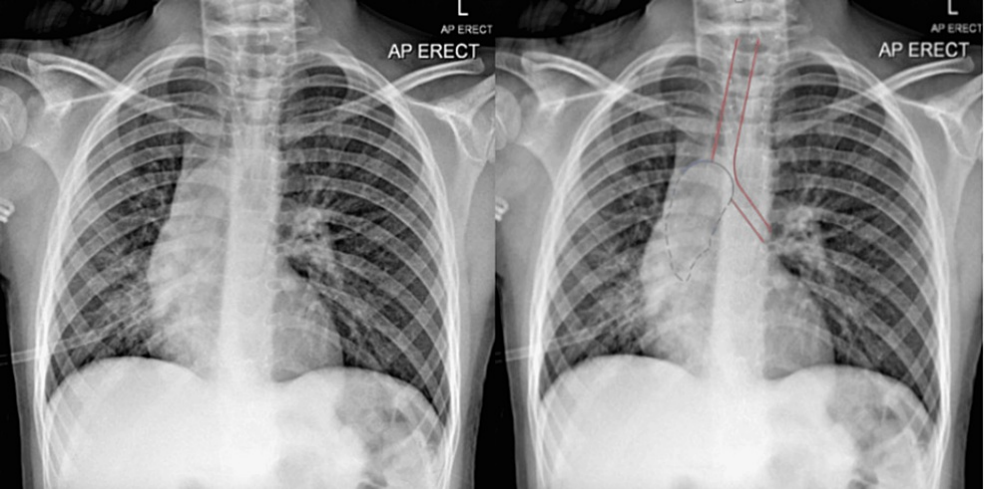
Left side: Presenting chest x-ray (CXR). Right side: CXR annotated; Solid red line: trachea and left main bronchus. Solid blue line: tumour projecting into distal trachea at the carina. Interrupted blue line: distal atelectasis and airways plugging behind the endobronchial tumour

On the repeat presentation, a CXR showed continuous consolidation to the right lower lobe (RLL). The child was given a further two-week course of antibiotics, which she completed at home. A follow-up CXR (see Figure 1), after discharge, demonstrated a hyperinflated left side, possible tracheal shift, and loss of volume of the right middle lobe (RML) and RLL. The case was then referred at this time to our centre for further management.

**Figure 2:**
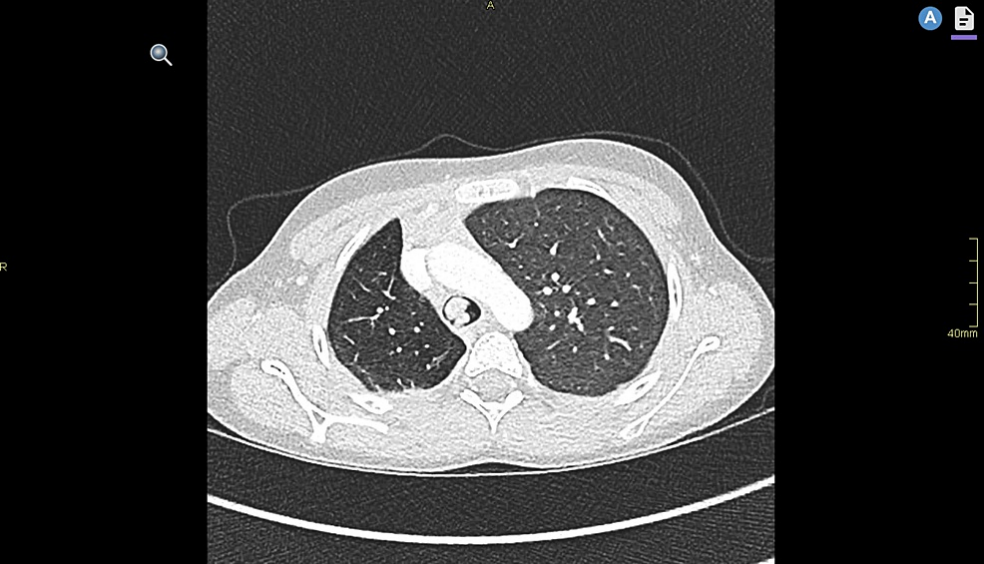
CT thorax axial section with contrast demonstrating a lobulated mass occupying much of the tracheal cross-sectional area

A computed tomography (CT) scan of the chest (Figure 2) was performed, which demonstrated a large lobulated soft tissue mass extending from the carina and right main bronchus and into the distal trachea, causing a significant reduction in the tracheal luminal area and was associated with RML and RLL obstructive bronchiectasis, plugging, and collapse. The mass demonstrated bright internal vascular enhancement. The mass was noted to be in close proximity to the azygos vein with no appreciable fat plane.

The differential diagnosis at this point was particularly MEC, inflammatory myofibroblastic tumour, chondrosarcoma, and carcinoid, with the enhancement and lobulation favouring the former.

**Figure 3:**
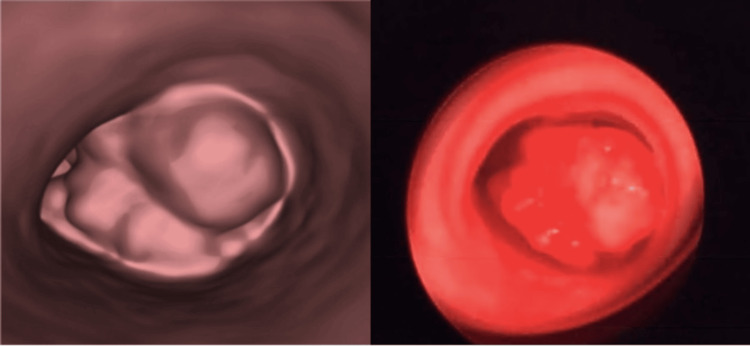
Left: CT virtual bronchoscopy image demonstrating the near-occlusive tumour (visualised from above). Right: Bronchoscopic image of tumour (visualised from above)

**Figure 4:**
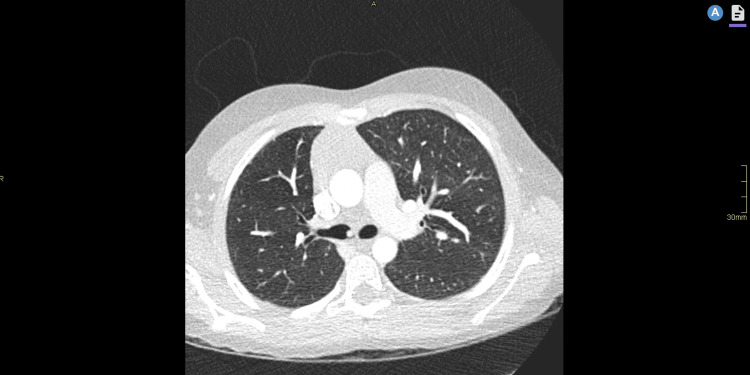
CT post debulking with a tiny enhancing stalk of residual tumour in the proximal right main bronchus

Subsequent to CT findings, the patient was promptly admitted to our institution for the management of her airway obstruction. Rigid bronchoscopy was performed, and an exophytic mass of approximately 80% of the total tracheal luminal area was noted to be arising from the junction of the right main bronchus and bronchus intermedius, which was occluding the RML and RLL and was crossing partially to the left airway without a complete occlusion (Figure 3). Multiple biopsies were taken, and the tumour was debulked. After the procedure, the child’s symptoms improved. Histological examination showed a low-grade MEC. The patient underwent a repeat bronchoscopy during the same admission, where further debulking was performed to achieve a patent right airway. She was discharged home and remained asymptomatic. A repeat CT scan several weeks later reported a significant improvement, in which the tumour had decreased in volume and the right lung had re-expanded (Figure 4).

The case was discussed in a multidisciplinary team (MDT) meeting, and the consensus was to offer definitive surgical treatment. The patient underwent a rigid bronchoscopy, right thoracotomy, carinal resection, right upper lobectomy, and frozen section three weeks after her initial bronchoscopy. 

Surgery

**Figure 5:**
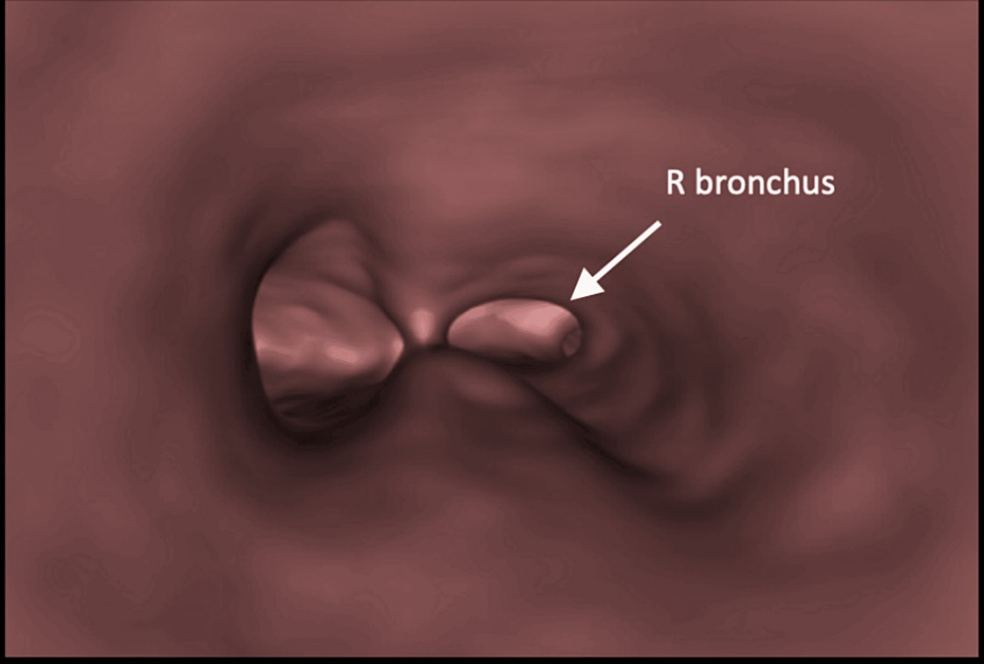
CT virtual bronchoscopy following right upper lobectomy and sleeve reconstruction

Via a right thoracotomy in the fifth intercostal space, the lung and bronchus were inspected. There was a significant portion of the right bronchus involved with associated fibrosis and contracture. Therefore, a hilar release procedure was completed to facilitate superior access. Dissection of the right bronchus intermedius, carina, and right upper lobe bronchus was undertaken, where the margins found grossly to involve the proximal part of the upper lobe required an upper lobectomy. This was followed by ligation of the azygous vein and systematic nodal dissection. Resection of the carinal wall of the left main bronchus was performed, and a reconstruction of the carina and bronchial wall was achieved. A neocarina was constructed by anastomosing the distal right bronchus intermedius and left main bronchus using interrupted 4-0 PDS sutures. Anastomosis of the distal tracheal lumen and neocarina was performed using 4-0 PDS sutures (Figure 5).

Intraoperatively, the frozen section demonstrated no evidence of malignancy in the sampled lymph nodes, and the right upper lobe margin edge was free of tumour as was the main bronchial resection margin. 

The anastomosis was assessed endobronchially with fibreoptic bronchoscopy, which showed satisfactory results. A pedicled intercostal interposition flap was wrapped around the reconstruction site for additional support. The patient tolerated the procedure well and was transferred to paediatric intensive care unit (PICU) for recovery.

Histopathology

Full histopathological analysis of the resection demonstrated that the exposed tumour measured 16x7mm. Sections from the specimen highlight the bronchus wall to be infiltrated by an MEC, with morphological features in keeping with diagnostic biopsy. The tumour showed solid, cystic, and glandular growth patterns, set in the fibrous stroma with occasional calcifications. There was a patchy moderate chronic inflammatory host response with the formation of tertiary lymphoid structures. The degree of nuclear pleomorphism was mild, necrosis was not a feature, and mitoses were not easy to identify. The tumour demonstrated some focally infiltrative growth but predominantly a pushing growth pattern into underlying fat. Lymph nodes demonstrated no granuloma formation or evidence of malignancy. The general conclusion is one of a low-grade, completely excised MEC with appropriate margins. Tumour staging as carina was involved by tumour: pT4 R0 N0.

Outcome and Follow-Up

A flexible bronchoscopy on postoperative day (POD) three demonstrated healthy anastomotic integrity. The child was weaned to room air by POD four and chest-drain and paravertebral blocks were removed on POD five, after which she was stepped down to the ward. She was discharged home on POD eight. 

Subsequent check bronchoscopies at four weeks, eight weeks, and six months were unremarkable, indicating successful airway reconstruction and resolution of the RML obstruction. Following the MDT discussion, there is no role for adjuvant therapies at present. The patient's symptoms have improved postoperatively, and she is currently under regular follow-up with yearly bronchoscopies for monitoring and surveillance.

## Discussion

Endobronchial MEC is a rare malignant tumour occurring in the glandular tissue of the bronchial submucosa and accounts for 0.1-0.2% of all primary lung cancers [1,2]. Reports on the sex incidence and age of MEC vary [6]. Of those cases described in children, many presenting symptoms are typical of airway obstruction consisting of wheezing, persistent cough, and recurrent chest infections [7]. Therefore, the labelling of “asthma” is not uncommon in these patients [8]. The generic nature of symptoms in the paediatric population associated with endobronchial pathology can be diagnostically difficult and often lead to delays or misdiagnosis. This, in addition to the rarity of endobronchial MEC, further compounds the time to diagnosis and, therefore, treatment.

The diagnostic workup of endobronchial MEC in children aside from CXR typically involves cross-sectional imaging studies such as CT or MRI. Radiographic findings typical of this condition involve air trapping with overinflation, regional collapse, and consolidation. CT findings can be nonspecific, though endobronchial MEC is typically seen as a distinct ovoid or lobulated intraluminal mass [9]. Endobronchial lesions are normally amenable to bronchoscopy, biopsy, and often debulking, with histopathological examination being fundamental to diagnosis [10].

MEC comprises squamous, mucus-secreting, and intermediate cells [3,4]. They are classified as high or low grade based on their histologic appearance. Low-grade malignancy exhibits fewer mitoses, nuclear pleomorphism, and necrosis, but with more cysts compared to their high-grade counterparts [11]. Local invasion is common; however, haematological spread is rare [12].

Surgical resection, particularly in low-grade tumours, is the treatment of choice with an excellent prognosis [13]. In high-grade cases, adjuvant treatment such as chemotherapy or radiation remains controversial [14]. The extent of surgery depends on several factors including the size of the tumour, location, and involvement of local structures. In appropriate cases, a sleeve resection may be possible; however, often a lobectomy or pneumonectomy is necessary to achieve a surgical cure [13]. There have been a small number of cases in the literature describing a novel endoscopic approach to the resection of low-grade endobronchial MEC with mixed results, with one of four cases presenting recurrence [15]. The goal of surgical resection is to achieve complete tumour removal while preserving lung function.

The long-term prognosis for paediatric patients with endobronchial MEC varies depending on the stage of the tumour, the presence of metastasis, and the adequacy of surgical resection. Regular follow-up with imaging studies and bronchoscopies is recommended to monitor for recurrence and evaluate treatment outcomes [16].

## Conclusions

We present a complex case of a seven-year-old girl with an endobronchial MEC causing RML obstruction, successfully managed through a combination of rigid bronchoscopy, right thoracotomy, carinal resection, right upper lobectomy, and reconstruction. This case highlights the high index of suspicion required to promptly identify endobronchial pathology and the importance of a multidisciplinary approach and surgical expertise in the lung-preserving treatment of paediatric patients with obstructive bronchial tumours.
